# Placental expression of estrogen and progesterone receptors and vascular endothelial growth factor in buffaloes suffering from uterine torsion

**DOI:** 10.1186/s12917-024-04422-z

**Published:** 2025-02-14

**Authors:** Yahia A. Amin, Ahmed Abdou Elnegiry, Eatemad A. Awadalla, Hassan A. Hussein, Ragab H. Mohamed

**Affiliations:** 1 Department of Theriogenology, Faculty of Veterinary Medicine, Aswan University, Aswan, 81528 Egypt; 2 Department of Cytology and Histology, Faculty of Veterinary Medicine, Aswan University, Aswan, Egypt; 3 Department of Zoology, Faculty of Science, Aswan University, Aswan, 81528 Egypt; 4 Department of Theriogenology, Faculty of Veterinary Medicine, Assiut University, Assiut, 71526 Egypt; 5 Faculty of Veterinary Medicine, Sphinx University, New Assiut, Egypt

**Keywords:** Uterine torsion, Placenta, Estrogen receptors, Progesterone receptors, Vascular endothelial growth factor, Buffaloes

## Abstract

**Background:**

Although several risk factors have been suggested for uterine torsion, the pathogenesis is still unclear. Therefore, the current study aims to investigate the pathogenesis of uterine torsion by assessing the histological, histochemical, and immunohistochemical changes that occur in the placenta obtained from uterine torsion cases. Immunohistochemical changes include investigation of the expression of estrogen receptors (ERs), progesterone receptors (PRs), and the vascular endothelial growth factor (VEGF) in the placental tissue.

**Methods:**

Forty intrapartum dairy cows were included in this investigation. The cows were divided into two equal groups. The first group was the uterine torsion (UT) group, while the second group was the normal control group (Ctrl). After caesarian section treatment, placentas were collected from all animals in the study. Histopathological, histochemical, and immunohistochemical examinations were performed. Estrogen receptors, progesterone receptors, and vascular endothelial growth factor expression in the placenta were evaluated.

**Results:**

The results revealed numerous trophoblast giant or binucleate cells in the trophoblastic epithelium. Through Masson’s trichrome technique, the distribution of collagen fibers as shiny, blue-colored stripes on the fetal mesenchyme was observed. Additionally, the results showed a strong, intense PAS-positive reaction in the cytoplasmic vesicles of most trophoblastic cells due to mucopolysaccharides. The immunohistochemical findings of the UT placenta revealed moderate to weak staining for ERs in contrast to those of the Ctrl placenta, which revealed moderate staining for ERs. In addition, non-statistical differences in the expression of PRs were found between the two tested groups. For VEGF, strong positive immunoreactivity was found in the Ctrl group compared to the UT group, which exhibits a general absence in many trophoblast cells.

**Conclusion:**

It can be concluded that significant variation was observed in the placentas obtained from buffaloes suffering from UT compared to those obtained from normal pregnant ones. These significant variations were involved in the decreased expression of ERs and VEGF in the UT group compared to the normal Ctrl one. Investigating the expression of these placental molecules may monitor the changes in the placental tissue and provide insight into the pathogenesis of UT.

## Introduction

Uterine torsion (UT) has been reported to occur in most domesticated species. However, due to variations in the strength of the uterine muscles, the location and distribution of the multiple ligaments supporting the uterus, and the mesenteric suspension in different species, uterine torsion is more common in some species than in others [1]. Asian countries account for most reported cases of uterine torsion [2].

The risk of UT originates from its consideration as a significant contributor to cow [3] and buffalo [4] dystocia, which endangers the life of the dam and the fetus. Uterine torsion has been observed in multiparous (81.7%) and primiparous (18.3%) buffaloes; it occurs throughout late pregnancy (58.4%) and in full-term (41.6%) buffaloes. Moreover, UT was found to induce fetal and maternal mortalities at a variant rate, as 78.6% were recorded for the fetal type and 23.8% were stated for the maternal type. Pregnancy stage and fetal viability were significant risk factors for maternal mortality, while the degree and duration of uterine torsion were important risk factors for fetal mortality [5].

During late pregnancy in cattle, uterine torsion can endanger the survival of the fetus and result in significant inflammatory and degenerative lesions in the uterus [6]. The duration and degree of torsion rotation are both factors that may expose the uterus to impaired uterine perfusion. Uterine congestion and cyanosis are prevalent because vascular twisting and constriction predominantly impact the veins. Therapy must be administered quickly to prevent the injured uterus from being deprived of blood, which will cause the vessels to thrombose and cause significant tissue alterations in the uterine wall.

Pathogenesis is still unclear, but several risk factors have been suggested. In cattle, the anatomy of the genital tract, single pregnancies [7, 8], age, breed, parity, housing, abdominal capacity due to high concentrate rations, behavior, and fetal weight [8, 9] are all factors identified to influence the occurrence of uterine torsion.

Retention of the placenta is one of the most common complications that usually occur after dystocia and thus has the potential to be studied nowadays [10–13]. Recently, the retained placenta was observed in at least 87% of the cases of postpartum uterine torsion [14]. The placenta is the only organ with a structural diversity comparable to other organs [15, 16]. Additionally, the placenta is a rich source of signal molecules that could significantly impact the placenta and the maternal or fetal compartment. These signal molecules in the placental tissue are responsible for its endocrine function [17].

Numerous mammalian species, particularly primates and ungulates, have a substantial amount of estrogen produced by the placenta. The development of the mammary glands during late gestation, myometrial excitability at the end of gestation, and the preparation of the birth canal for parturition have all been linked to the effects of placental estrogens in cows [17]. Furthermore, progesterone (P4) is the primary hormone responsible for the maintenance of pregnancy. During pregnancy, the placenta in cows slightly influences the levels of total progestogens in mothers, mostly luteal. However, rather than a decrease in placental progesterone production, evidence of undiminished placental P4 tissue concentrations and 3β-hydroxysteroid dehydrogenase activities till term [18] suggests a higher demand for P4 throughout late gestation. Placental progesterone may be crucial for producing high local concentrations at the feto-maternal interface, which may require some concentration-dependent progesterone effects. However, this is especially true for species whose luteal P4 synthesis is the predominant mode of P4 synthesis throughout gestation [19]. In any other case, placental P4 might only be a byproduct or intermediary of the production of different steroids, particularly estrogens.

Angiogenesis plays a crucial role in the implantation process and the substantial (30–50 times) increase in uterine blood flow during pregnancy in female reproductive organs, such as the uterus, corpus luteum, and placenta [20, 21]. Preeclampsia, intrauterine growth restriction, and pregnancy loss are linked to disturbances in uterine vascular development [22].

It is well known that the twisted uterus’s restricted blood supply causes hypoxia, ischemia, and necrosis, which irreversibly harm the endometrium and myometrium and ultimately result in the fetus’s death [23, 24]. Furthermore, uterine wall elasticity and viability are lost due to ongoing blood supply failure, making the wall necrosed, fragile, brittle, and prone to rupture [25]. Activating angiogenesis is one of the most effective ways to increase the oxygenation of hypoxic tissue to counteract these detrimental effects [26]. Vascular endothelial growth factor (VEGF), commonly referred to as vascular permeability factor, is one of the factors that can alter angiogenesis. It is the most accurate indicator of a particular regulator of endothelial cell development and differentiation [27]. In addition, it was previously reported that a successful pregnancy depends on sufficient VEGF expression [28, 29], and modifications to the VEGF system may result in placental dysfunction.

From all the above, this study hypothesizes that UT induces variant changes in the placental tissue. These changes could be detected by investigating the common signaling molecules in the placenta. The most common signaling molecules in the placental tissues are placental E2, P4, and VEGF. Therefore, the current study aims to investigate the expression of estrogen receptors (ERs), progesterone receptors (PRs), and the VEGF in the placental tissue obtained from UT buffalo cases. Investigation of the expression of these molecules may monitor the changes that occur in the placental tissue and provide insight into the pathogenesis of UT.

## Materials and methods

### Animals

All procedures were carried out according to the guidelines that were approved by the ethics committee of the Faculty of Veterinary Medicine, South Valley University, Egypt (approval number (VM/SVU/24(7)-04)) and the ethics committee of the Faculty of Science, Aswan University, Egypt (approval number (ASWU/05/SC/ZO/24 − 07/R.29)). The current study was carried out on buffaloes that were presented for treatment in the clinics of obstetrics and gynecology, Faculty of Veterinary Medicine of Aswan University. A total of 40 intrapartum baladi breed pluriparous dairy buffaloes were included in the current study; twenty suffered from uterine torsion. While the normal control group (Ctrl) contains the same number and gave birth without suffering from uterine torsion or uterine pathological condition. All included buffaloes at the time of presentation were in due time of parturition, where the owner complained that the buffaloes showed signs of labor and straining began without progress of calving, especially in the group that suffered from uterine torsion where the cesarean sections for them were decided.

When the animal was referred, all signs of approaching parturition, such as slackening of the broad pelvic ligaments, vulva edematization, enlargement and edema in the udder, and erected teats, were noticed. In some cases, the drop of the mucus cervical seal and the appearance or ruptures of the water bags (especially the Ctrl group) were recorded, and in most cases, the owners gave the time of mating or insemination. Vaginal and transrectal examinations were used to confirm the existence of uterine torsion and to ascertain its degree and direction [30]. The duration of uterine torsion was defined as the period from the diagnosis of delayed calving until the cesarean section was applied. To be included in the uterine torsion group, the animals had to have a rectal body temperature below 39 °C, maintain standing ability, and not have undergone any attempts at retorsion before referral to the clinic [31]. Cesarean sections were applied in the group that suffered from uterine torsion only. Calving for the Ctrl group should be spontaneous, or only moderate obstetrical assistance (simple fetal extraction) could be performed for termination of the parturition process without difficulties. In addition, upon examination, the genitalia and general health of these animals were found to be normal. Directly after calving (Ctrl group) and during cesarean section (a group with uterine torsion), specimens of the fetal membranes were collected to be examined for histopathological, histochemical, and immunohistochemical investigation.

### Histological examinations

After being collected, placenta fragments were cleaned in sterile saline and preserved in 10% neutral phosphate-buffered formalin (pH = 7.0). Specimens were cleaned in methyl benzoate, gradually dehydrated in 50–99% ethyl alcohol, and then embedded in molten paraffin wax at 58–62 °C for microscopic preparations. Single sections with a thickness of 5 μm were cut using a microtome, and the collagenous fibers were stained with Masson trichrome and Harris’s hematoxylin and eosin stain.

### Histochemical studies for general carbohydrates

The periodic acid Schiff (PAS) approach was used for this purpose. In this case, 0.5% periodic acid was used first to oxidize the carbohydrates. However, this released the aldehyde groups (-HCO-HCO) from the glycol groups (-HCOH-HCOH), which subsequently reacted with Schiff’s reagent to produce a molecule that was colored pink.

### Immunohistochemical investigations

#### Expression of estrogen receptors, progesterone receptors and VEGF

The demonstration of ERs, PRs, and VEGF expressions in the placenta was carried out using the AB clonal kit method (ABclonal Technology Company, USA). Formalin-fixed, paraffin-embedded placenta tissue slices were sectioned, 4–5 μm thick. The sections were deparaffinized and rehydrated. After applying the antigen retrieval solution, the endogenous peroxidase was inactivated for 15 min using 3% hydrogen peroxide, and then the solution was blocked for an hour. The rabbit polyclonal primary antibodies (5% normal goat serum diluted in PBS/TBS supplemented with 0.3% Triton™ X-100, pH = 7.2) with catalog number (A12357 for ERs, A0321 for PRs, and A0280 for VEGF) were applied to each section and incubated overnight in a humidified chamber at 4ºC. Then, after applying the peroxidase-labeled secondary antibody [HRP goat-anti-rabbit IgG (H + L)] with catalog number (AS014) at a 1:200 dilution, the samples were incubated for 30 min. Freshly prepared DAB substrate and chromogen were used at room temperature for 2 to 5 min. Haematoxylin was used as a counterstain; sections were dried and then mounted.

Histological and immunological alterations were examined under an Olympus BX43F high-power light microscope, Tokyo 163–0914, Japan. A personal computer, a camera, software (Olympus DP74 Tokyo 163–0914, Japan), and an optical microscope were used for the image analysis.

### Histomorphometric study and image analysis

Histomorphometric analysis was used to assess the quantification of structural changes determined by the histological analysis of placental tissues. After placental tissues were histologically processed, digital images were taken under the objective lens magnification of 40X using a digital camera connected to a light microscope. Morphometric analysis was performed using a computerized image analysis software, Image J version 6. Spatial calibration with an object micrometer was performed before each analysis. Five pictures were selected from each animal in each group. The following morphometric parameters were measured: the thickness of the villus, collagen fiber percent (intensity/surface area), polysaccharide percent (content/surface area), and expression intensity/surface area of ERs, PRs, and VEGF.

### Statistical analysis

The data from the placental measurements were expressed as the mean value (mean ± SEM). The t-test (SPSS, Inc., 2020) was used to assess the significant difference between the UT and Ctrl groups (the level of significance was set at *P* < 0.01 and/or 0.001).

## Results

### Histological findings

**Fig. 1:**
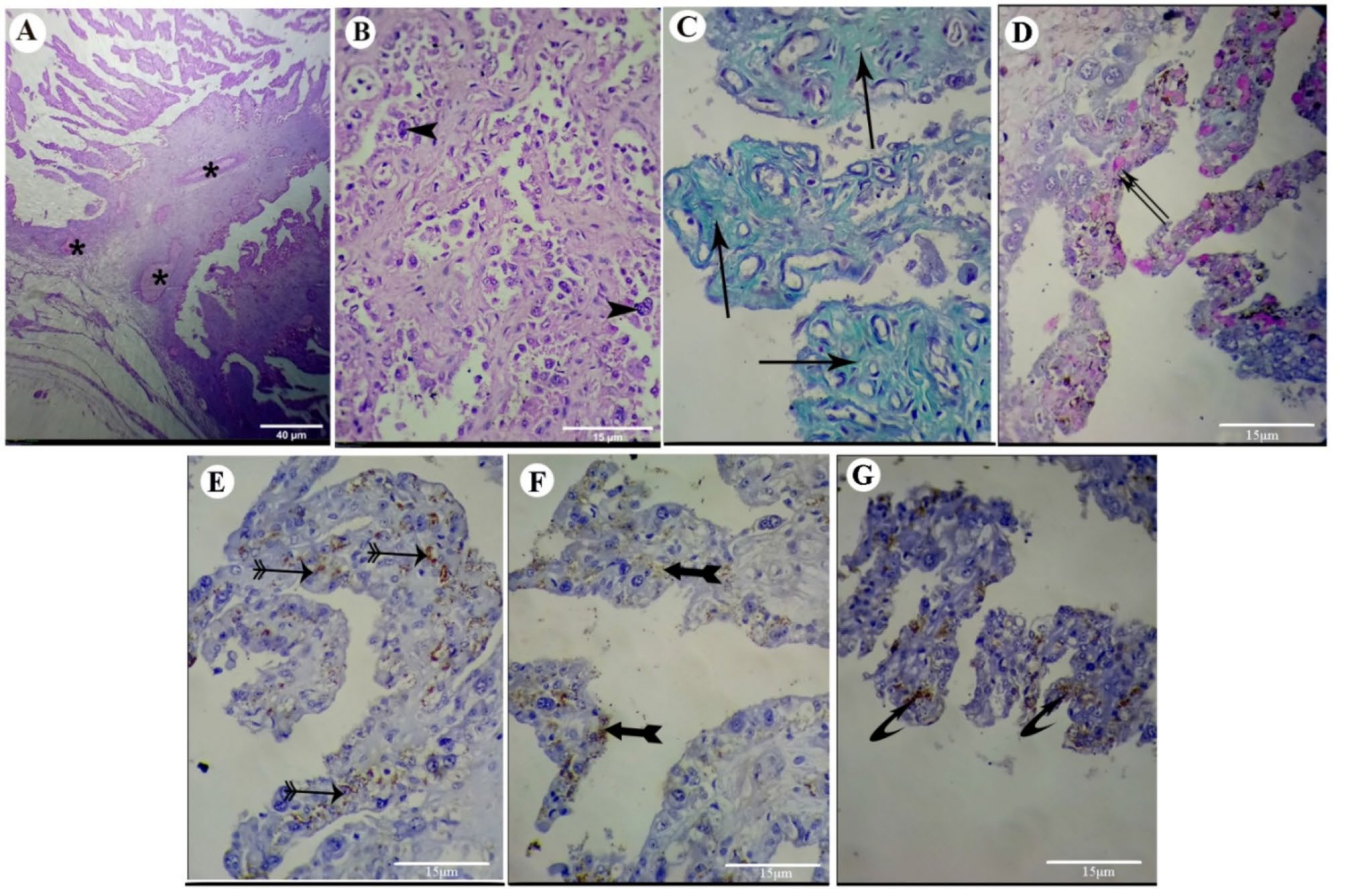
(**A**) to (**G**): photomicrographs of placental tissue of the control group. (**A**) and (**B**): Photomicrographs of normal placenta stained with H&E. (**C**): photomicrograph of the placenta stained with Masson’s trichrome stain. (**D**): photomicrograph of the placenta stained with PAS reagent. (**E**): photomicrograph of the placenta prepared for ERs immunostaining. (**F**): photomicrograph of the placenta prepared for PRs immunostaining. (**G**): photomicrograph of the placenta prepared for VEGF immunoreactivity. Blood vessels (stars), binucleate cells (arrow head), collagenous fibers (arrow), mucopolysacharides inside the cytoplasmic vesicles (double arrows), moderate immunoreactivity to the estrogen receptors (archery arrows ↣), weak positive immunoreactivity to the PR receptors (forked tail arrow ➼), and marked positive VEGF immuno-reactivity (curved arrow ⤤). H&E: haematoxylin and eosin; PAS: periodic acid-Schiff; ERs: estrogen receptors; PRs: progesterone receptors; VEGF: vascular endothelial growth factor. [Original magnification: (**A**): 40 μm, (**B**) to (**G**): 15 μm]

The control placental section, stained with hematoxylin-eosin, showed several forms of branched chorionic villi forming secondary and tertiary villus. Every villus comprises vascular mesenchyme, with trophoblastic cells covering it (Fig. 1A). A vascular mesenchyme with elongated connective tissue cores that was much more vascularized with blood vessels was additionally observed. Some blood vessels were located adjacent to the epithelium. Numerous trophoblast giant cells, or binucleate cells, could be visualized in the trophoblastic epithelium. These cells had vesicular nuclei and lightly stained cytoplasm (Fig. 1B).

**Fig. 2:**
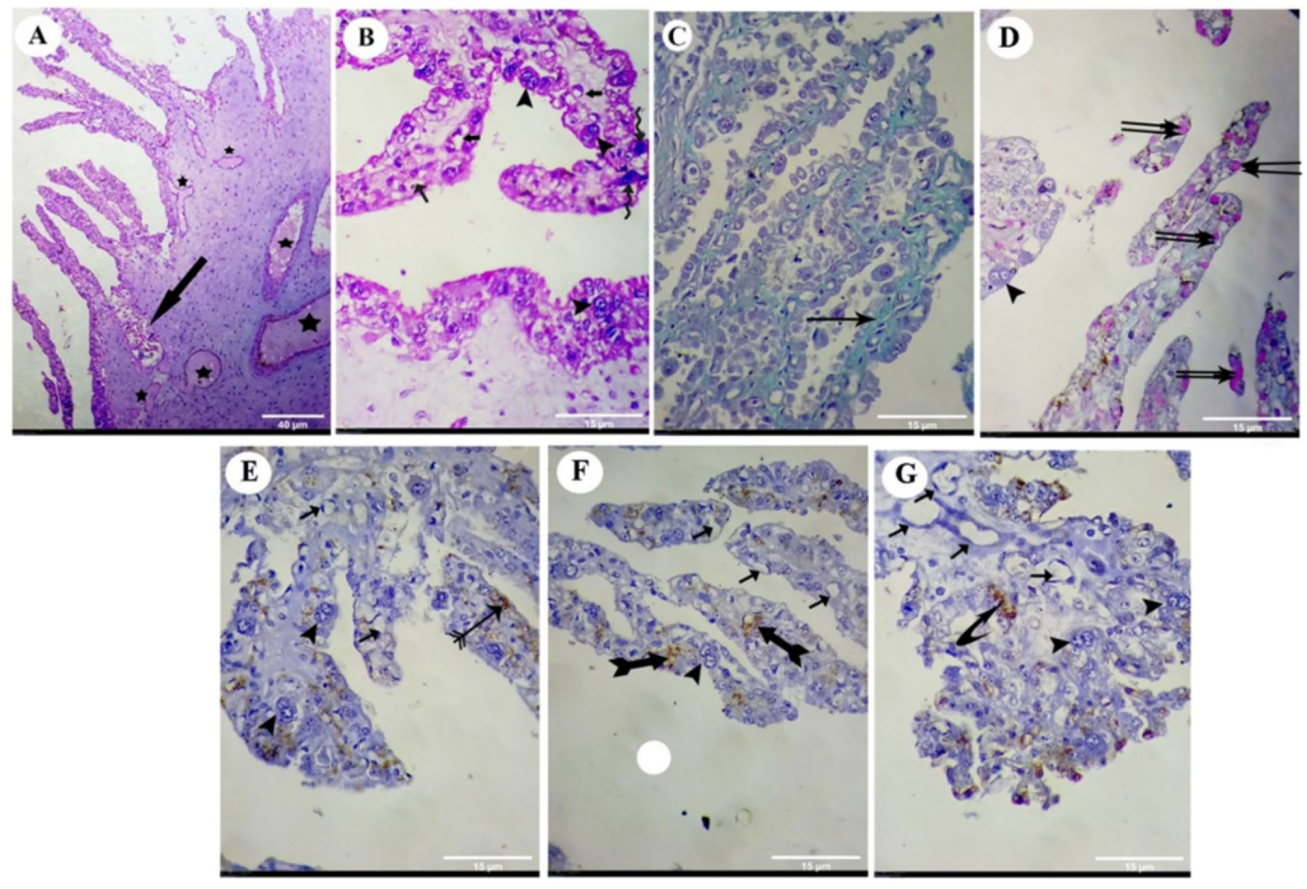
(**A**) to (**G**): photomicrographs of the placenta of the uterine torsion. (**A**) and (**B**): photomicrographs of the placenta stained with H&E. (**C**): photomicrograph of the placenta stained with Masson’s trichrome stain. (**D**): photomicrograph of the placenta stained with PAS reagent. (**E**): photomicrograph of the placenta prepared for ERs immunostaining. (**F**): photomicrograph of the placenta prepared for PRs immunostaining. (**G**): photomicrograph of the placenta prepared for VEGF immunoreactivity. Inflammatory infiltration (thick arrows), blood vessel congestion (stars), binucleate cells (arrow head), cytoplasmic vacuolization (small arrow), collagenous fibres (arrow), mucopolysacharides inside the cytoplasmic vesicles (double arrows), weak immunoreactivity to the estrogen receptors (archery arrows ↣) and the progesterone receptors (forked tail arrow ➼), a small number of trophoblast cells express VEGF (curved arrow ⤤). H&E: haematoxylin and eosin; PAS: periodic acid-Schiff; ERs: estrogen receptors; PRs: progesterone receptors; VEGF: vascular endothelial growth factor. [Original magnification: (**A**): 40 μm, (**B**) to (**G**): 15 μm]

**Fig. 3:**
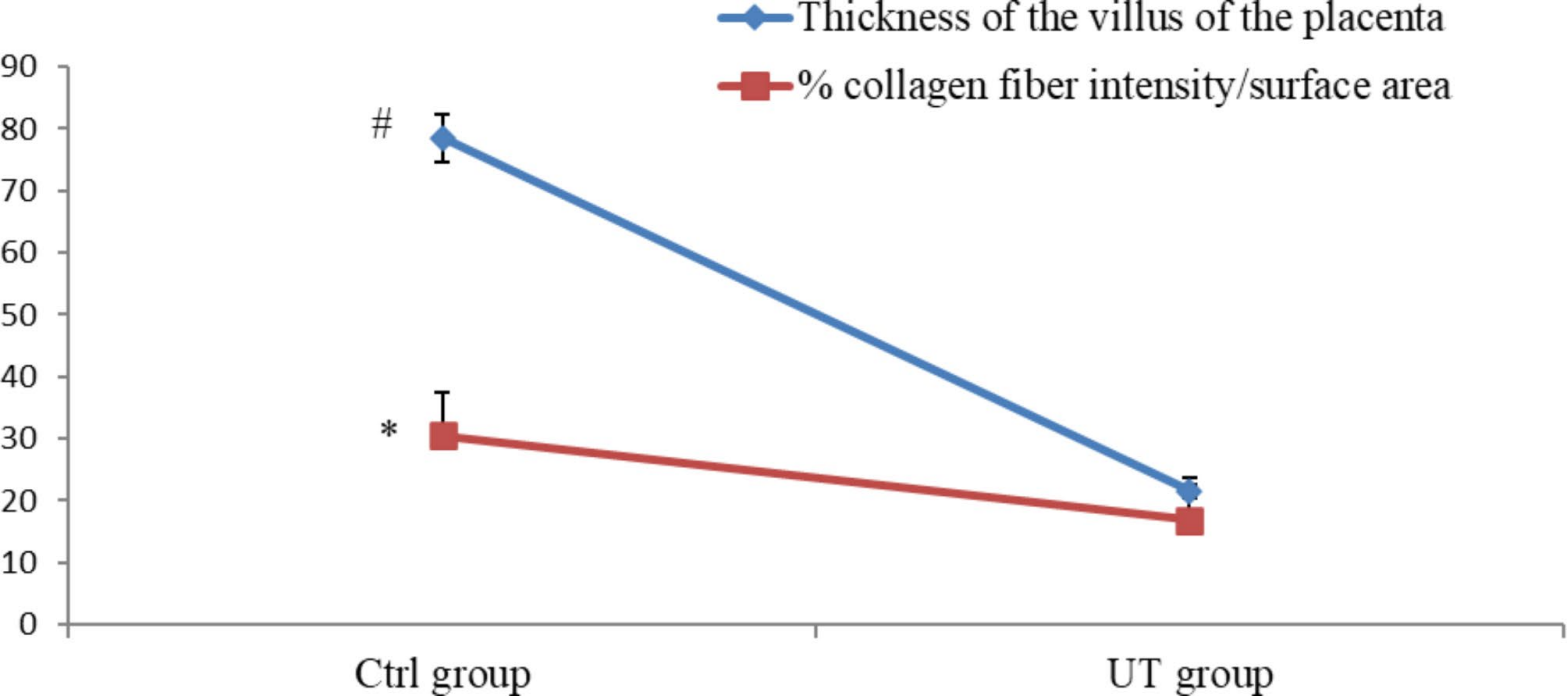
Mean ± SEM of the morphometric analysis of the placental sections in ctrl and UT group of buffaloes: Thickness of the villus of the placenta and percent (%) of collagen fiber intensity/surface area. Value with superscript (* and #) differ significantly on the same line at *p* < 0.001. Ctrl-group: control group, UT- group: uterine torsion group

The placental features of the UT group were consistent with severe inflammatory infiltrations between the chorionic villi and congestion of blood vessels. In addition to inflammatory changes, the placenta revealed microcotyledonary atrophy. This atrophy was characterized by short, small villi with a narrowed base (Fig. 2A). Comparison between the UT group and the Ctrl group revealed a statistically significant decrease (*P* < 0.001) in the thickness of the villus of the placenta of the UT group compared to the Ctrl group (Table 1; Fig. 3). Moreover, the UT group’s placenta displayed necrotic microcotyledons in the chorionic cells. Binucleated cells exhibited features of degeneration, such as irregular cellular and nuclear morphology. Furthermore, there was intense cytoplasmic vacuolization in the trophoblastic cells of chorionic villi (Fig. 2B). 

**Table 1:** Mean ± SEM of the morphometric analysis of the thickness of the villus, percent (%) collagen fiber intensity/surface area, percent (%) polysaccharide content/surface area, percent (%) ERs expression intensity/surface area, percent (%) PRs expression intensity/surface area and percent (%) VEGF expression intensity/surface area of both control and UT- group sections

Measurements	Ctrl- group	UT- group
Thickness of the villus	78.51 ± 1.93	21.57 ± 0.42 ^a^
% collagen fiber intensity/surface area	30.53 ± 2.08	16.90 ± 1.11 ^a^
% polysaccharide content/surface area	5.77 ± 0.38	2.92 ± 0.29 ^a^
% ERs expression intensity/surface area	6.42 ± 0.97	2.85 ± 0.18 ^b^
% PRs expression intensity/surface area	4.91 ± 0.30	3.88 ± 0.42
% VEGF expression intensity/surface area	6.85 ± 0.53	3.36 ± 0.35 ^b^

SEM: Standard error mean; Ctrl-group: control group, UT- group: uterine torsion group. ERs: estrogen receptors, PRs: progesterone receptors and VEGF: vascular endothelial growth factor

a Significantly different from the control group at *p* < 0.001

b Significantly different from the control group at *p* < 0. 01

Masson’s trichrome technique revealed that the distribution of collagen fibers appeared as shiny blue-colored stripes at the fetal mesenchyme in the Ctrl group (Fig. 1C). In contrast, the same technique in the UT group showed that the collagenous fibers were stained lightly blue at the fetal mesenchyme (Fig. 2C). Comparison between the UT group and Ctrl group revealed a statistically significant decrease (*P* < 0.001) in the percent of collagen fiber intensity/surface area in the UT group compared to the Ctrl group (Table 1; Fig. 3).

### Histochemical results

**Fig. 4:**
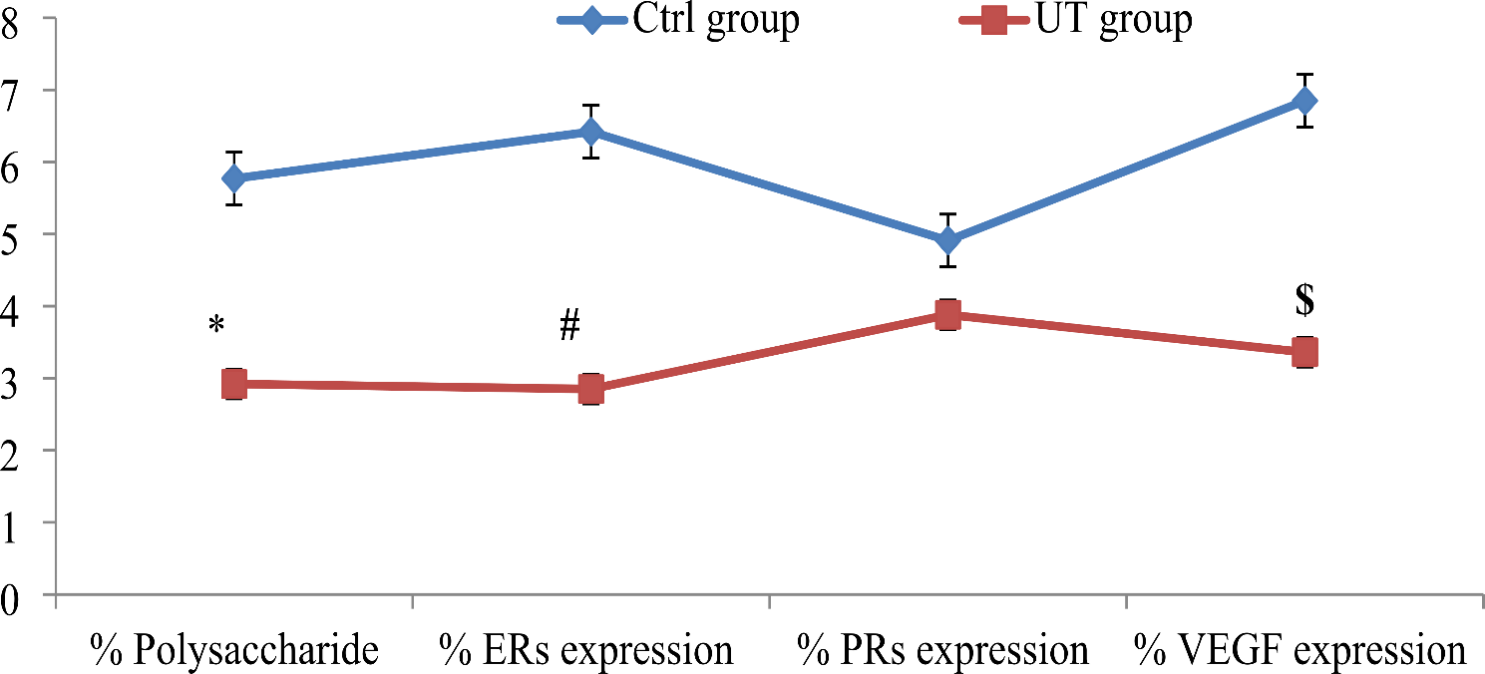
Mean ± SEM of morphometric analysis of the placental sections in ctrl and UT group of buffaloes: percent (%) of the polysaccharide content/surface area, percent (%) of the ERs expression intensity/surface area, percent (%) of PRs expression intensity/surface area and percent (%) of the VEGF expression intensity/surface area. Values of the superscripts (*, # and $) at different lines differ significantly. Value with superscript (*) differ significantly at *p* < 0.001, while values of the superscripts (# and $) differ significantly at *p* < 0.01. Ctrl-group: control group, UT- group: uterine torsion group. ERs: estrogen receptors, PRs: progesterone receptors and VEGF: vascular endothelial growth factor

The PAS reaction in the placenta of the control group revealed an intense positive reaction in the cytoplasmic vesicles of most trophoblastic cells due to mucopolysaccharides (Fig. 1D). In contrast, the reaction in the placenta of the UT group showed a few chorionic villi trophoblastic cells with a positive PAS stain (Fig. 2D). Comparison between the UT group and the Ctrl group revealed a statistically significant decrease (*P* < 0.001) in the percentage of polysaccharide content/surface area in the UT group compared to the Ctrl group (Table 1; Fig. 4).

### Immunohistochemical results

The control placenta revealed moderate ERs expression staining and weak PRs expression in most trophoblast cells of the chorionic villi (Fig. 1E and F, respectively). In addition, the cytotrophoblast cell layer surrounding the fetal villi showed strong positive VEGF expression, while the villous stromal cells revealed no immunoreactivity (Fig. 1G).

The placenta of the UT group showed moderate to weak staining for the ERs and PRs expressions in most trophoblastic cells of chorionic villi (Fig. 2E and F, respectively). The VEGF expression of the placenta of the UT group was generally absent in most trophoblastic cells, except a few trophoblastic cells that express VEGF (Fig. 2G).

Comparisons between the two groups revealed a significant decrease in the percent of ERs expression intensity/surface and the percent of VEGF expression intensity/surface area in the UT group compared to the Ctrl group (*P* < 0.01) (Fig. 4). In contrast, a non-significant difference was observed in the PR expression intensity/surface percentage between the two groups (Table 1; Fig. 4).

## Discussion

In the present study, the histological examination of the placenta of the Ctrl group revealed that the placental tissue was normal and well nourished by blood supply, while in the UT group, the placenta displayed atrophy, necrosis, and congestion of blood. Previous studies reported that UT was found responsible for cell death and irreversible damage to the myometrium and endometrium, which contribute to the uterine wall becoming necrosed, brittle, fragile, and prone to rupture [1, 25].

In the current research, the histologic features of the placenta from the UT group showed severe inflammatory infiltrates between the chorionic villi, congestion of the blood vessels, necrosis, and atrophy of the microcotyledons in the chorionic cells. These results are in line with those stated in the previous studies. McEntee [32] reported that uterine torsion is associated with inflammatory changes and infection that can cause adhesions to surrounding abdominal tissues. In addition, Malik [33] demonstrated that the endometrial epithelial lining was typically absent, the myometrium exhibited various degenerative patterns, the uterine muscles were worn out, ruptured, and hemorrhagic, and the endometrium displayed congestion, hemorrhages, and occasional adhesions.

In the current research, numerous trophoblast giant cells, or binucleate cells, could be visualized in the trophoblast epithelium in the Ctrl group. These cells had vesicular nuclei and lightly stained cytoplasm. In contrast, in the placenta of the UT group, binucleated cells exhibited features of degeneration, such as irregular cellular and nuclear morphology, which could be referred to as blood supply deprivation and oxygen deficiency, which led to its necrobiotic changes and death. It was previously mentioned that the placentas of ruminant animals contain binucleate cells in the fetal trophectodermal epithelium that are essential for creating the feto-maternal interface’s structures and secretions, which may be vital in causing and sustaining pregnancy [34]. However, this may explain the end of pregnancy and the death of the fetus in most UT animal cases.

The placenta is a transient endocrine organ responsible for hormone production, such as estrogen. The cotyledons, the fetal section of the placentome, are the sites of estrogen (E2) synthesis. These sites are where the trophoblast large cells contain aromatase (CYP19A1), the primary enzyme in the manufacture of estrogen [35, 36]. It is still unclear how critical placental estrogens are during pregnancy’s first and middle phases. They probably work as a growth and differentiation factor for the uterus or placenta [17, 36].

In the current research, the immunohistochemical findings of the UT placenta revealed moderate to weak staining for ERs in the majority of trophoblast cells of chorionic villi, which is in contrast to the immunohistochemical findings of the Ctrl placenta, as the latter revealed moderate staining for ERs. It was reported in a previous study that intrapartum UT has a negative impact on placental E2 synthesis and/or release into the maternal circulation, which significantly lowers peripheral E2 levels [37]. These results suggest that even with effective obstetric therapy, reduced placental estrogens and other regulatory factors present in the maternal compartment may lead to compromised uterine function during the subsequent puerperium. In addition to the disruption of uterine perfusion leading to inadequate synthesis of placental estrogens or their transfer to the maternal compartment, it is possible that uterine torsion could be facilitated by a primary disruption of placental E2 and prostaglandin production. This disruption may result in decreased myometrial contractility, causing the uterus to become flaccid and more susceptible to uterine torsion. Unfortunately, we could not confirm this because we did not analyze PG concentration, indicating a need for further investigation.

In the current research, the immunohistochemical findings of the UT and Ctrl placenta revealed non-marked differences in the expression of P4 in the majority of trophoblastic cells of chorionic villi, as the same weak staining of PRs was found in the UT and Ctrl groups. Similar findings were reported in a recent study conducted on intrapartum UT cows in Turkey, in which the P4 concentrations did not differ statistically between the UT and Ctrl groups [37].

In the peripheral maternal circulation, most P4 that is detectable throughout gestation is always of ovarian origin, even though the placenta of cows shows notable concentrations of P4 in placental tissue from local production starting around 180 days of pregnancy [36]. There was no discernible bovine uterine contribution to maternal P4 levels during the final stage of gravidity [38]. Therefore, it is unclear how a relationship may be established between the extremely varied amounts of free estrogens and the basal or slightly suprabasal P4 concentrations at birth.

In the current study, in the placenta of the Ctrl group, the cytotrophoblast cell layer surrounding the fetal villi showed strong positive VEGF immunoreactivity, while the villous stromal cells revealed no immunoreactivity. In contrast, the VEGF immunoreactivity findings of the placenta of the UT group were generally absent in most trophoblast cells, except for a few trophoblastic cells.

Little information is available about the role of VEGF in the placenta of the animal. Most research about VEGF is concerned with its expression in humans. The current study’s findings are similar to those previously stated in human studies. According to these studies, women who have recurrent pregnancy loss (RPL) have considerably lower levels of VEGF expression in their decidua and chorionic villi than do women who have normal pregnancies [39, 40]. Vascular endothelial growth factor inhibits vascular endothelium cell apoptosis and facilitates angiogenesis [41]. It was stated that VEGF contributes to trophoblast cell migration, proliferation, and endovascular differentiation [42, 43]. Therefore, disruption of the VEGF system may result in placental dysfunction.

Furthermore, it has been demonstrated that blocking VEGF signaling through VEGFR inhibition causes a reduction in luteal endothelial networks, vascular endothelial cell detachment, luteal steroid-producing epithelial cell death, disturbed luteal function, cessation of embryonic development, and preterm birth [44, 45]. Nevertheless, this may explain the exposure of most uterine torsion cases to the death of the embryo.

In the current research, the ERs display weak staining in the UT group compared to the Ctrl group. Estrogen receptors are expressed in uterine arterial endothelial cells (ECs) [46], as well as in other types of ECs [47], which proposes that 17β-estradiol (E2β) can act directly on the cells and alter uterine vascular function. According to reports, E2β can increase the number of endothelial progenitor cells (EPCs), which in turn can boost vasculogenesis [48] and angiogenesis [49, 50]. The weak expression of ERs in the UT group may explain the weak staining of VEGF in the UT group compared to the Ctrl group, which indicates that a decrease in ERs expression results in a reduction of VEGF in the UT buffaloes. However, a previous study stated that one possible explanation for dysregulated VEGF expression could be genetic variation [51].

## Conclusion

Uterine torsion decreases the expression of ERs and VEGF in the buffalo uterus, suggesting a possible role in the pathogenesis of uterine torsion. However, further research is warranted to fully understand the pathogenesis of such pathology and its threads in severe cases.

## Data Availability

Data availability statements.
